# Mechanisms of Cellular Membrane Reorganization to Support Hepatitis C Virus Replication

**DOI:** 10.3390/v8050142

**Published:** 2016-05-20

**Authors:** Hongliang Wang, Andrew W. Tai

**Affiliations:** 1 Division of Gastroenterology, Department of Internal Medicine, University of Michigan Medical School, Ann Arbor, MI 48109, USA; wanghon@med.umich.edu; 2 Department of Microbiology and Immunology, University of Michigan Medical School, Ann Arbor, MI 48109, USA; 3 Medicine Service, Ann Arbor Veterans Administration Health System, Ann Arbor, MI 48105, USA

**Keywords:** viral replication, double membrane vesicle, RNA virus, phosphatidylinositol 4-phosphate, phosphatidylinositol 4-kinase

## Abstract

Like all positive-sense RNA viruses, hepatitis C virus (HCV) induces host membrane alterations for its replication termed the membranous web (MW). Assembling replication factors at a membranous structure might facilitate the processes necessary for genome replication and packaging and shield viral components from host innate immune defenses. The biogenesis of the HCV MW is a complex process involving a concerted effort of HCV nonstructural proteins with a growing list of host factors. Although a comprehensive understanding of MW formation is still missing, a number of important viral and host determinants have been identified. This review will summarize the recent studies that have led to our current knowledge of the role of viral and host factors in the biogenesis of the MWs and discuss how HCV uses this specialized membrane structure for its replication.

## 1. Introduction

Hepatitis C virus (HCV) is a globally prevalent human pathogen. More than 170 million people are chronically infected worldwide, among whom many will develop cirrhosis and hepatocellular carcinoma. HCV is an enveloped, single-stranded positive-sense RNA virus classified in the *Hepacivirus* genus within the *Flaviviridae* family. The 9.6 kb genome contains one open reading frame (ORF) that is flanked by non-translated regions, which are necessary for viral RNA translation and replication [1,2,3]. A single polyprotein is translated from the ORF, which is co- and post-translationally processed by cellular and viral proteases to generate ten mature proteins: Core, E1, E2, p7, NS2, NS3, NS4A, NS4B, NS5A, and NS5B. The structural proteins (Core, E1, and E2) are incorporated into virus particles, whereas the nonstructural proteins p7 to NS5B coordinate the intracellular processes of the virus life cycle [1,2,3]. While p7 and NS2 are dispensable for genome replication, they are required for particle assembly [4,5]. NS3 through NS5B are necessary and sufficient for HCV genome replication. As we will discuss extensively in this review, HCV replication is associated with the induction of host membrane alterations that are thought to support sites of viral RNA replication. The induction of altered host membranes for viral replication is characteristic of all positive sense RNA viruses [6]. A negative sense replicative intermediate synthesized from the positive sense RNA genome serves as template for the generation of progeny positive sense RNA genomes. The newly-synthesized positive sense RNA can either enter a new translation/replication cycle or be packaged into virions [2]. This review will summarize our current knowledge on HCV-induced membrane alterations, as well as the role of viral nonstructural proteins and host factors in this process.

## 2. History and Methods of Studying the HCV Membranous Web

Even before the identification and molecular cloning of hepatitis C virus, electron microscopy (EM) studies of liver tissue from chimpanzees infected with “non-A, non-B hepatitis” demonstrated membrane alterations in hepatocytes [7,8]. The successful isolation of the first HCV cDNA clone [9] enabled studies to determine the effects of expressing viral proteins in hepatocytes in cell culture. Egger *et al.* reported that expression of the entire HCV polyprotein in U2-OS human osteosarcoma cells was associated with the formation of membrane alterations described as vesicles within a membranous matrix, which collectively was termed “membranous webs” (MWs) [10]. In this study, expression of NS4B alone also induced membrane alterations similar to those seen with the whole viral polyprotein.

**Figure 1:**
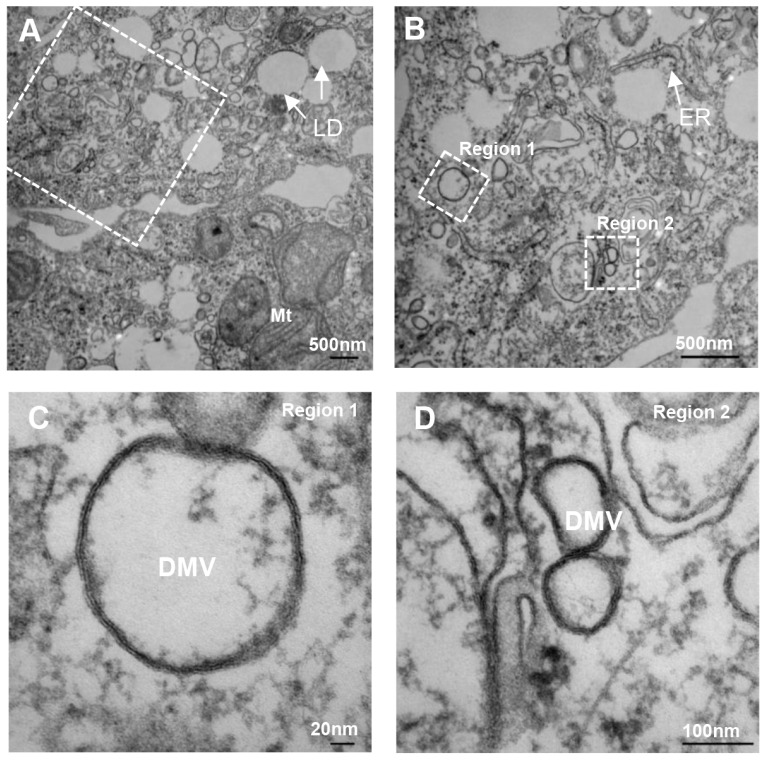
Ultrastructure of membranous webs and double-membrane vesicles (DMVs). (**A‒D**) Huh 7.5.1 cells were infected with the JFH1 strain of hepatitis C virus (HCV) for 54 h before fixation and processing for transmission electron microscopy. Consecutive enlargements of the boxed areas are shown. “Region 1” and “Region 2” in panel (**B**) are shown in panels (**C** and **D**), respectively. ER: endoplasmic reticulum; Mt: mitochondria; LD: lipid droplets.

The subsequent establishment of the replicon model of HCV replication made it possible to visualize HCV induced membrane alterations in the context of viral genome replication. By using cell lines harboring persistent subgenomic replicons, Gosert *et al.* [11] found altered membrane structures similar in ultrastructural morphology to those observed by Egger *et al.* [10]; furthermore, by using immunogold EM, the authors reported that the membranous web could be labeled by antibodies against each of the HCV nonstructural proteins. At the light microscope level, immunofluorescence labeling for NS3 or NS5A was visible as dot-like cytoplasmic structures [11,12,13,14]. A later study of cells containing a subgenomic HCV replicon reported that the membrane alterations induced by HCV replication consisted in part of double-membrane vesicles (DMVs; Figure 1) with a diameter around 200 nm that were also positive on immunoelectron microscopy with antibodies against NS5A and double-stranded RNA (dsRNA) [15]. This was a notable observation, as a number of other RNA viruses also induce DMVs in infected cells [6,16].

The morphology of MWs described above was confirmed and extended by using the cell culture infectious, full-length Jc1 clone of HCV in conjunction with high pressure freezing with freeze substitution and electron tomography [17]. In this study, Romero-Brey *et al.* found that HCV infection was initially associated with accumulation of DMVs with an average diameter of 150 nm. The kinetics of DMV accumulation correlated with viral RNA replication; at later time points of infection, multi-membrane vesicles (MMVs) with larger diameter (~330 nm) became more predominant [17]. 3D reconstructions of EM tomographic series showed that most of the DMVs were tightly opposed to ER membranes, and some of them were identified as protrusions from the ER membrane into the cytosol, suggesting that MWs originate from ER membranes [17]. In addition, most of the DMVs were closed structures, with only a minority possessing either a visible opening towards the cytosol or a short neck-like structure connecting the DMV to the ER membrane bilayer [17].

The identification of sites within HCV proteins that tolerate the insertion of heterologous sequences, such as epitope tags and even fluorescent proteins (for example, domain III of NS5A tolerates the insertion of green fluorescent protein (GFP)), made it possible to study the dynamics of MW in live cells. NS5A-GFP was found in the cytoplasm as brightly fluorescing dots and in a reticular staining pattern [18], similar to the distribution of NS5A observed in fixed and immunostained replicon cells. Live cell imaging revealed two populations of NS5A-GFP foci in cells: larger, relatively static structures and smaller structures with saltatory microtubule-dependent movements over long distances, both of which contain other HCV replicase components [19]. However, the relationship of the two structures to one another is not well understood; for example, it is not known whether the smaller structures are precursors of or arise from larger static structures, or whether either structure participates preferentially in genome replication *versus* particle assembly. Another approach to visualizing HCV replication organelles dynamics employed SNAP (a mutant of the human DNA repair protein O6-alkylguanine-DNA alkyltransferase) tagging of NS5A [20], which permitted labeling of temporally-distinct populations of NS5A. This approach revealed that NS5A synthesized 48–72 h before imaging was located on structures distinct from those associated with NS5A synthesized 0–48 h before imaging, which provides an upper bound for the duration of viral polyprotein translation at a given replication organelle.

A fundamental shortcoming of light microscopy is its limited resolution. Correlative light electron microscopy (CLEM), which integrates the molecular specificity of fluorescent light microscopy with the resolution of EM, has been employed to study the ultrastructural morphology of MWs [17] and has also been used to analyze the effect of NS5A small molecule inhibitors [21] or NS5A mutants [22] on the morphology of MWs.

The imaging techniques to study MW morphology and dynamics summarized above have been complemented by biochemical studies. Unfortunately, *in vitro* reconstitution of a functional HCV replicase is still beyond our technical reach. Early studies showed that crude membrane fractions isolated from HCV subgenomic replicon cells or selectively permeabilized replicon cells could use endogenous replicon RNA as a template to synthesize new viral RNA [12,14,23,24,25,26]. Viral RNA synthesis was found to be resistant to protease and nuclease treatment, suggesting that replication occurs in membrane structures [12]. Additionally, membrane fractions containing HCV RNA and nonstructural proteins were found to have properties similar to detergent-resistant membranes (DRMs) in that they were resistant to solubilization by cold Triton X-100 and that these DRMs co-fractionated with cellular markers of DRMs on density gradient centrifugation [14,26]. The specialized lipid and protein composition of the HCV replication organelle might, therefore, facilitate the generation of membrane curvature necessary for DMV formation [27]. In addition, as DRMs are important platforms for cellular membrane trafficking and signal transduction [27,28], it is conceivable that this property of HCV replication membranes may also regulate signal transduction and membrane trafficking at viral replication sites.

While the viral RNA is largely resistant to nuclease treatment, only a small fraction (2%–3%) of viral NS protein is resistant to protease treatment [26,29]. A likely explanation for this observation is the large excess of HCV protein compared to positive- and negative-strand HCV RNA; indeed, a quantitative analysis of HCV RNA and protein content of replicon cells estimated that there is a 1000-fold excess of HCV protein over HCV RNA [29].

In addition to subcellular fractionation, attempts have been made to isolate MWs or replicase components by affinity capture of tagged NS proteins [30,31,32] or viral RNA with associated proteins [33,34]. One of these studies using epitope-tagged NS4B identified DMVs in the affinity-purified fraction and the presence of replicase activity associated with DMVs [30], providing direct evidence that DMVs are a site of HCV RNA synthesis.

## 3. Biogenesis of the Membranous Web

Although HCV has long been known to induce membrane rearrangements, it is only recently that some of the mechanisms that are responsible for the formation of these structures have begun to be unraveled. Despite the substantial progress that has been made during the past few years, we are still far from understanding this complex process in detail. In the following sections, we summarize what is known about the role of both viral and cellular proteins in HCV-induced membrane reorganization.

### 3.1. Role of Viral Factors in Membranous Web Biogenesis

By using HCV polyprotein overexpression, Egger *et al.* [10] first showed that expression of viral nonstructural proteins resulted in membrane alterations that morphologically resemble those observed in replicon cells, demonstrating that viral RNA replication is not required for membranous web formation. By expressing individual HCV proteins, these authors found that expression of NS4B was sufficient to induce membrane alterations resembling MWs. Later studies have shown that the N-terminal alpha helix AH2 [35] as well as C-terminal sequences [36] are important for HCV replication, NS4B oligomerization, and DMV morphogenesis. Mutations of either of the two positively-charged lysine residues flanking the N-terminal alpha helix AH1 abrogate HCV replication and are associated with the formation of significantly larger DMVs when expressed in the context of NS3-5B using a non-replicative system [37].

Subsequent studies, however, have provided evidence that NS5A is the only HCV NS protein capable of forming DMVs when expressed in isolation [17,22], albeit much less efficiently than when expressed in the context of NS3-5B. In contrast, expression of NS4B alone leads to the exclusive formation of single-membrane vesicles rather than DMVs [17]. The amino-terminal “domain 1” of NS5A is necessary and sufficient for the formation of DMVs when expressed in the context of the NS3-5B polyprotein [22]. This work also identified roles of other HCV NS proteins in efficient DMV formation, notably the NS3 helicase domain, and the expression of the NS3-4A protease *in cis* with NS4B-5B. Finally, mutations that accelerate the normally slow polyprotein cleavage kinetics at the NS4B-5A junction or constructs that do not express any NS4B-5A precursor impair or abrogate DMV formation [22], suggesting that a NS4B-5A precursor is somehow essential for DMV biogenesis. Further evidence for a functional interaction between NS4B and NS5A comes from the identification of mutations in NS5A that rescue mutations flanking NS4B AH1 [37] or help rescue a NS4B C-terminal mutant [36]. Overall, these studies suggest that most, if not all, nonstructural proteins are required to work in concert for the efficient formation of DMVs.

### 3.2. Roles of Host Factors in Membranous Web Biogenesis

In addition to viral proteins, an increasing list of host factors has also been shown to contribute to membranous web formation. Here, we well briefly discuss the mechanisms of several selected host factors in MW formation.

#### 3.2.1. PI4KA—PI4P—OSBP and FAPP2: Cholesterol and Glycosphingolipids

Several RNA interference screens have identified the cellular lipid kinase PI4KA (also known as phosphatidylinositol 4-kinase III alpha, PI4KIIIα, and PIK4CA) as essential for HCV replication [38,39,40,41,42]. Inhibition of PI4KA, either by RNA interference [40,42,43,44] or by pharmacologic inhibitors [45], leads to accumulation of large ‘clusters’ of NS5A-positive membranes at the light microscopic level. At the ultrastructural level, these ‘clusters’ correspond to clusters of DMVs with reduced diameter [42], suggesting that PI4KA is essential for the proper formation and/or integrity of MWs. NS5A and NS5B can interact with PI4KA and NS5A activates its lipid kinase activity, giving rise to elevated intracellular phosphatidylinositol 4-phosphate (PI4P) levels [42,43]. PI4P has a highly negatively-charged headgroup and has been reported to cause membrane curvature at physiologically relevant concentrations [46], so local production of PI4P at nascent replication organelles might facilitate membrane curvature and DMV formation. Another function of PI4P is to recruit specific viral and/or host proteins with PI4P-binding domains [47]. In particular, two PI4P effectors, oxysterol-binding protein (OSBP) and four-phosphate adaptor protein 2 (FAPP2) are essential for HCV replication [32,48], and inhibition of either OSBP or FAPP2 results in altered MW morphology [48,49]. Interestingly, OSBP and FAPP2 are both lipid transfer proteins (LTPs), which are responsible for non-vesicular sterol and glycosphingolipid trafficking, respectively [50]; both of these lipids are important components of DRMs generally and, likely, also of HCV replication organelles specifically. Inhibition of OSBP leads to reduced trafficking of cholesterol to HCV replication organelles [49]. Similarly, the LTP ceramide transfer protein CERT has also been reported to be involved in the HCV life cycle [51]. These findings suggest a model in which PI4P recruits LTPs such as OSBP and FAPP2 to HCV replication organelles, which in turn result in the trafficking of cholesterol and glycosphingolipids to HCV replication membranes.

#### 3.2.2. Membrane Deforming Proteins

DMVs are highly-curved structures; it is likely that proteins and/or lipids with membrane-deforming properties are involved in MW biogenesis. One such protein with membrane-deforming activity, proline-serine-threonine phosphatase-interacting protein 2 (PSTPIP2), has been identified as a host factor essential for HCV viral replication and MW formation [52]. PSTPIP2 is a member of the Pombe Cdc15 homology (PCH) family proteins with membrane-deforming properties, likely mediated by their F-BAR domain. PSTPIP2 co-fractionates with detergent-resistant membranes regardless of the presence of HCV, interacts with both NS4B and NS5A, and co-localizes with NS5A on MWs by immunoelectron microscopy. Mutations in PSTPIP2 predicted to ablate its membrane-deforming function rendered it less effective in rescuing HCV replication in cells silenced for endogenous PSTPIP2 relative to expression of wild-type protein.

Another member of the PCH family, bridging integrator 1 (BIN1), has been reported to be possibly involved in the HCV life cycle through an interaction with NS5A [53], though it is not known whether BIN1 is essential for viral replication or participates in MW biogenesis. The precise contribution of PCH family proteins and other membrane-deforming proteins to MW formation remains to be determined.

#### 3.2.3. Nuclear Pore Complex Proteins

As a positive-sense RNA virus, HCV is not known to require the nucleus for any step in its infection cycle. However, putative nuclear localization signals and nuclear export signals have been reported in HCV proteins (reviewed in [54]), and some reports have described localization of core protein and NS5A to the cell nucleus during viral infection [55,56,57,58]. Furthermore, HCV and other positive-sense RNA viruses appear to interact with nucleocytoplasmic transport factors [59]. More specifically, HCV infection has been reported to directly interact with and relocate nuclear transport components, including karyopherins and nucleoporins, to sites enriched for HCV replication and assembly [60]. Furthermore, knockdown of a few of these karyopherins and nucleoporins impairs viral replication and/or virion assembly [60]. These findings raises the intriguing hypothesis that relocation of nuclear transport components to the HCV replication organelles might influence membrane curvature and/or transport factors across membranes of DMVs and other replication organelle structures. However, this remains to be functionally demonstrated.

#### 3.2.4. Autophagy and DMV Formation

Autophagy is a cellular response to a variety of stimuli, including nutrient depletion, hormone treatment, and viral or bacterial infection in eukaryotic cells [61]. One of the most distinguishing features of autophagy is the formation of double-membrane vesicles called autophagosomes, which engulf cytoplasmic macromolecules and damaged organelles and deliver them to lysosomes for degradation and recycling. While the cellular origin of the autophagosome membrane is not completely established, the endoplasmic reticulum may be one of its membrane sources [62]. Given these similarities between cellular autophagosomes and the DMVs seen in HCV infection, multiple studies have examined the possibility that DMV formation in HCV infection exploits the cellular autophagocytic machinery (reviewed in [63]).

Several studies have reported that the expression of HCV replicons e.g., [15,64] or HCV infection [65] induces the accumulation of autophagosomes in cultured cells. Other studies have demonstrated that ectopic expression of HCV NS4B or NS5A is also sufficient to induce autophagic vesicles and upregulate markers of autophagy induction, such as lipidated LC3 [66,67], though whether these findings reflect effects of protein overexpression is unclear. In addition, an important question that arises from these experiments is whether autophagy and autophagosome induction are byproducts of HCV infection or whether autophagy itself is necessary for HCV infection and replication.

While multiple studies have indicated that autophagy is somehow important for productive HCV infection, there is controversy regarding the precise steps of HCV infection that are facilitated by autophagy. Several groups have reported that autophagy plays an important role in HCV RNA replication [15,68,69,70,71]. However, Dreux *et al.*, found that autophagy specifically modulates the onset of translation of incoming HCV RNA and, therefore, the initial establishment of HCV replication [72], and this observation was supported by another study [69]. In addition to a possible role for autophagy in establishing HCV replication, Tanida *et al.* observed that the release of HCV core and infectious particles from infected cells is reduced when autophagy is inhibited, and they proposed that in addition to facilitating the initiation of viral replication, autophagy proteins also contribute to HCV particle assembly and/or egress [73]. We still do not understand the molecular mechanisms by which the autophagy machinery supports either of these processes; in particular, why autophagy should be required only for the establishment of HCV replication but not for the maintenance of ongoing replication remains to be elucidated.

#### 3.2.5. Models of Membranous Web Formation

The biogenesis of membranous web is a complex process involving a concerted effort of HCV nonstructural proteins and a growing list of host protein and lipid factors. Twenty-seven years after the molecular cloning of HCV, we all still far from understanding the molecular processes that lead to MW formation in the HCV-infected cell. Based on our current state of knowledge, we will discuss candidate general mechanisms that direct MW formation.

**Figure 2:**
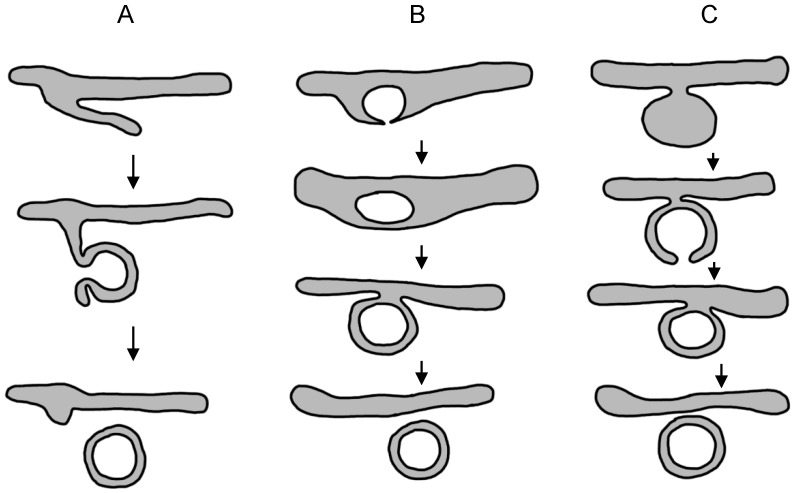
Possible models for the formation of virus-induced double-membrane vesicles from the endoplasmic reticulum. (**A**) Protrusion and detachment model; (**B**) double-budding model that begins with invagination into the ER lumen; and (**C**) tubulation and invagination model that begins with membrane exvagination from the ER.

It is generally believed that HCV induced MWs are derived primarily from the host cell ER membrane [10,15,17,18]. As MMVs appear later in HCV infection than DMVs, after the peak of HCV RNA replication [17], and as several other RNA viruses also induce the formation of DMVs, most investigators have focused on the mechanisms of DMV morphogenesis rather than on MMVs. Studies of other viruses that also induce the formation of ER-derived DMVs have led to the proposal of several models for the formation of virus-induced double-membrane vesicles, including but not limited to a protrusion and detachment model, a double-budding model, and a model of exvagination, followed by invagination [6,17,74] (Figure 2). These models are not necessarily mutually exclusive and, in theory, could operate simultaneously in infected cells. The first ‘protrusion and detachment model’ invokes local bending/deformation of part of an ER cisterna with tight apposition of the two lipid bilayers, followed by pinching off and sealing to form a double-membrane vesicle. In the ‘double budding model', a single-membrane vesicle buds by invagination into the ER lumen, from which it is subsequently released by a second budding event into the cytosol to give rise to a DMV. In the last model, exvagination or tubulation of the ER membrane is followed by partial invagination to form a cup-like structure that is then sealed to form a DMV. In the case of HCV, 3D reconstruction of electron tomographic images reveals that virus-induced DMVs are exvaginations connected via a short neck-like structure to the ER membrane bilayer, and most DMVs are linked to the ER only via their outer membrane [17].

At this point in time none of these models has been convincingly demonstrated to be a mechanism of DMV formation. However, kinetic analysis of the ultrastructural membrane alterations following HCV [17,75] infection have identified single-membrane vesicles early in HCV infection, while similar studies of enterovirus-infected cells have identified single-membrane tubules [76], which could be precursors of DMVs and thus might argue for models A and/or C presented above.

## 4. Functions of the Membranous Web

### 4.1. The Membranous Web and HCV Genome Replication

The membranous web appears to be the site of HCV RNA genome replication. By immunofluorescence microscopy, newly synthesized viral RNA metabolically labeled with 5-bromouridine 5’-triphosphate (BrUTP) co-localizes with NS5A protein as a marker of the membranous web [11,14,18]. Furthermore, negative strand HCV RNA, which is a necessary intermediate of HCV replication, has been detected at NS5A-positive foci by confocal microscopy [77].

The precise localization of the HCV replicase complex at the different membranous structures that make up the membranous web (e.g., SMVs, DMVs, and MMVs) has not yet been unequivocally determined, as localization of nascent HCV RNA or the HCV negative strand has yet to be clearly demonstrated at the ultrastructural level e.g., [17,28]. This may be due to poor accessibility of the interior of membrane structures to RNA labeling reagents and/or to incompatibility between EM sample preparation techniques that preserve ultrastructural detail and currently available RNA labeling methods. We will discuss three lines of evidence that HCV RNA replication occurs in association with DMVs.

First, immunoelectron microscopy using an anti-dsRNA monoclonal antibody suggests that dsRNA labeling is associated with DMVs in HCV-replicating cells [17,75] and with DMVs immunoisolated from HCV-infected cells [30]. While it is assumed that dsRNA-containing replicative intermediates are formed as a result of viral negative-strand RNA synthesis and thus indicative of sites of viral RNA replication, it is likely that some fraction of dsRNA-containing foci is not actively engaged in RNA synthesis and instead represents inactive replication complexes or products of replication.

Second, analysis of the kinetics of HCV RNA and DMV accumulation in acutely infected cells has shown a correlation between the two, while the appearance of MMVs lags significantly behind both [17], suggesting that DMVs might be the principal site of HCV RNA replication and that MMVs are not a major site of HCV RNA replication. There are two caveats to this interpretation: a role for single-membrane vesicles (SMVs) in HCV replication was not specifically evaluated in this study, and correlation between DMV and viral RNA accumulation does not exclude the possibility that DMVs serve as storage sites for replication-inactive HCV RNA molecules that have been synthesized elsewhere. A similar study of the kinetics of poliovirus-induced membrane alterations found that the appearance of SMVs correlated best with the exponential phase of viral RNA synthesis, while DMVs appeared only later in infection [78].

Third, as already mentioned above, perhaps the most direct evidence that DMVs are sites of HCV RNA synthesis is a study of replicon cells expressing epitope-tagged NS4B [28]. DMVs are presented in affinity-purified membranes from these cells, and about half of these DMVs can be labeled by BrUTP in *in vitro* replicase assays. However, the immunogold labeling of BrUTP was observed both on the exterior and in the interior of DMVs, leaving unresolved the question of whether the HCV replicase is located on the interior or on the exterior of the DMV.

In favor of the former model is the observation that HCV RNA is sensitive to nuclease digestion only in the presence of detergents e.g., [12,14,79], and this is also true of *in vitro* replicase activity of membranes isolated from HCV replicon cells [29]. On the other hand, replicase localization within a membrane-enclosed compartment raises the question of how ribonucleotides and other molecules necessary for RNA synthesis gain access to the replicase complex and how progeny RNA genomes exit the membrane structure. In an ultrastructural study using cryoelectron tomography of HCV-infected cells, only about 8% of all DMVs had an identifiable opening connecting the interior to the cytosol [17]. The outer membrane bilayer of 45% of DMVs was contiguous with the ER membrane, but the inner bilayer appeared to be closed. A spherical DMV of 125 nm in diameter [17,49], assuming a ribonucleoside tri-phosphate (rNTP) concentration of 5 mM [80] and no exchange with the cytosol, will contain only about 3000 molecules of each rNTP, which is enough to synthesize only about one complete HCV genome. Therefore, any model of HCV replicase localization within a DMV or membrane structure of comparable volume must also allow for a mechanism for rNTP replenishment. It may be that only the minority of DMVs with an opening to the cytosol are engaged in active genome replication. Another possibility is that protein channel(s) in both DMV membrane bilayers mediate the entry of rNTPs and other necessary factors, though this has not been experimentally demonstrated.

An alternative model is that the HCV replicase complex is located on the cytosolic surface of DMVs or another membrane compartment. This would be analogous to the poliovirus replicase complex, which is associated with the cytosolic face of virus-induced vesicles [81]. Coronaviruses also generate DMVs during infection [82,83]; the nonstructural protein 2 (nsp2) of murine hepatitis coronavirus has been shown to localize to the cytosolic face of DMVs [82]. Although this would appear to be inconsistent with the known nuclease resistance of HCV RNA and HCV replicase activity, it would resolve the problem of accessibility of rNTPs and other factors to the replicase complex.

### 4.2. Other Potential Functions of the Membranous Web

Membrane association of the HCV replicase complex has also been shown to shield viral RNA from innate immune recognition. The HCV RNA genome and its replicative intermediates are thought to encode potent pathogen-associated molecular patterns (PAMPs) recognized by host cell pattern recognition receptors (PRRs) such as retinoic acid-inducible gene 1 (RIG-I) and melanoma differentiation-associated protein 5 (MDA5) (reviewed in [84]). Recently, Neufeldt *et al.* showed that both RIG-I and MDA5 are excluded from the HCV replication organelles, and that addition of a nuclear localization signal to RIG-I or MDA5 resulted in their replicase complex localization and stimulation of immune response [85]. These results would appear to support a model of HCV replicase and viral RNA localization inside DMVs, and suggest that enclosure of the HCV replicase and HCV RNA within membranous structures restricts access of PRRs to HCV-encoded PAMPs and by doing so, protect viral RNA from innate immune recognition.

## 5. Future Perspectives

Despite important recent advances in our understanding of the molecular requirements of the HCV replication process, many important questions remain unresolved. It is still not clear how viral proteins and host factors work in concert to alter the host ER membranes to initiate the formation of MWs. It is also not known whether the replication of HCV occurs on the exterior or in the interior of DMVs. Advances in microscopy techniques, including cryo-electron tomography, focused ion beam scanning electron microscopy, and superresolution microscopy, will probably contribute to future breakthroughs in this field. Finally, while this review focuses on the replication of HCV, how genome replication is coordinated with polyprotein translation or virion assembly is not well known. As all of these are general questions shared by all positive-sense RNA viruses, answers to these questions will benefit not only the study of HCV but also of positive-sense RNA viruses.

## Abbreviations

The following abbreviations are used in this manuscript:

DMV: double-membrane vesicle
DRM: detergent resistant membrane
EM: electron microscopy
HCV: hepatitis C virus
MMV: multi-membrane vesicle
MW: membranous web
PAMP: pathogen-associated molecular pattern
PCH: Pombe Cdc15 homology
PI4P: phosphatidylinositol 4-phosphate
PRR: pattern-recognition receptor
SMV: single-membrane vesicle
